# JAK1-preferential inhibition in refractory inflammatory bowel disease: reframing upadacitinib as a strategy for rapid immune recalibration

**DOI:** 10.3389/fimmu.2026.1893892

**Published:** 2026-07-20

**Authors:** Xiaowei Liu, Yang Wang

**Affiliations:** Department of Thoracic Surgery, Central Hospital of Dalian University of Technology (Dalian Municipal Central Hospital), Dalian, China

**Keywords:** Crohn’s disease, immune recalibration, inflammatory bowel disease, JAK1-preferential inhibition, precision therapy, refractory IBD, ulcerative colitis, upadacitinib

## Abstract

Inflammatory bowel disease (IBD) management has moved from symptom control toward treat-to-target strategies, yet many patients with ulcerative colitis or Crohn’s disease remain difficult to treat after primary non-response, secondary loss of response, or intolerance to advanced therapy. Upadacitinib, an oral Janus kinase 1 (JAK1)-preferential inhibitor, has shown clinically meaningful efficacy in both ulcerative colitis and Crohn’s disease, including biologic-experienced populations. In this Perspective, we propose rapid immune recalibration as a hypothesis-generating clinical model for interpreting rapid inflammatory control following JAK1-preferential inhibition in selected patients with inflammation-dominant refractory IBD, rather than as an established biological mechanism or evidence of a durable immune reset. Recalibration is defined here as an early pharmacologically induced shift in the intensity and balance of convergent cytokine signaling, not as immune homeostasis restoration, mucosal healing, transmural repair, fibrosis reversal, or proven disease modification. We distinguish established clinical evidence from mechanistic rationale and from unvalidated downstream hypotheses, including barrier stabilization, microbiome change, anti-fibrotic potential, and biomarker-guided positioning. We also outline measurable clinical criteria, feasible biomarkers, exploratory translational endpoints, and safety boundaries that should guide future testing of this framework. This more bounded interpretation may help inform future efforts to move refractory IBD treatment from empirical drug switching toward mechanism-informed therapeutic choice without overstating the current evidence.

## 1 Introduction

The management of inflammatory bowel disease (IBD), including ulcerative colitis (UC) and Crohn’s disease (CD), has moved from symptom control toward treat-to-target care. This shift prioritizes objective control of inflammation, mucosal healing, and prevention of cumulative bowel damage, yet many patients remain difficult to treat after biologic failure or intolerance (1–3).

The conventional sequence of biologic switching often treats refractory disease as a problem of insufficient drug options. A more useful interpretation is that refractory IBD reflects biological heterogeneity. Some patients may have inflammation driven by redundant cytokine networks, whereas others may have disease dominated by immune-cell trafficking, microbial-immune interaction, tissue remodeling, or established structural damage. In this setting, the clinical value of an advanced therapy should be judged not only by efficacy rates, but also by the inflammatory signaling profile of the patient subgroup in which it works.

Upadacitinib is an oral, JAK1-preferential inhibitor that modulates intracellular signaling downstream of multiple cytokines. Its efficacy in UC and CD has established it as an important therapeutic option, but its broader conceptual significance should be framed carefully. In this Perspective, we propose rapid immune recalibration as a testable and hypothesis-generating framework: early pharmacological modulation of convergent JAK1-dependent signaling, attenuation of inflammatory amplification loops, and possible downstream stabilization of epithelial stress in selected inflammation-dominant refractory IBD. This framework does not imply universal superiority, cure, or proven long-term disease modification. Rather, it provides a bounded way to position JAK1-preferential inhibition in putative cytokine-driven inflammatory states and to define the clinical and safety boundaries of such an approach.

## 2 Defining rapid immune recalibration: scope and boundaries

Rapid immune recalibration is used here as a hypothesis-generating clinical model rather than as an established biological mechanism. The term describes an interpretive framework in which early symptom improvement, early biomarker changes, and objective inflammatory assessment are considered together to evaluate whether JAK1-preferential inhibition is producing rapid inflammatory control in selected patients with active inflammatory disease. In this sense, recalibration refers to a putative early shift in inflammatory signaling balance, not to a proven or durable immune reset.

This definition is intentionally narrower than immune restoration or disease modification. Recalibration does not mean cure, complete immune homeostasis, durable mucosal healing, transmural healing, fibrosis reversal, or structural modification. These outcomes require objective endpoints and longer follow-up, and they should not be inferred from symptom kinetics alone.

The concept also differs from general anti-inflammatory efficacy. Broad cytokine suppression describes a pharmacological effect, whereas rapid immune recalibration is proposed as a clinical interpretive framework linking early intracellular pathway modulation to measurable changes in inflammatory burden. The framework would be weakened or refuted if rapid symptoms were not accompanied by biochemical, endoscopic, histologic, radiologic, or molecular evidence of reduced inflammation.

**Figure 1:**
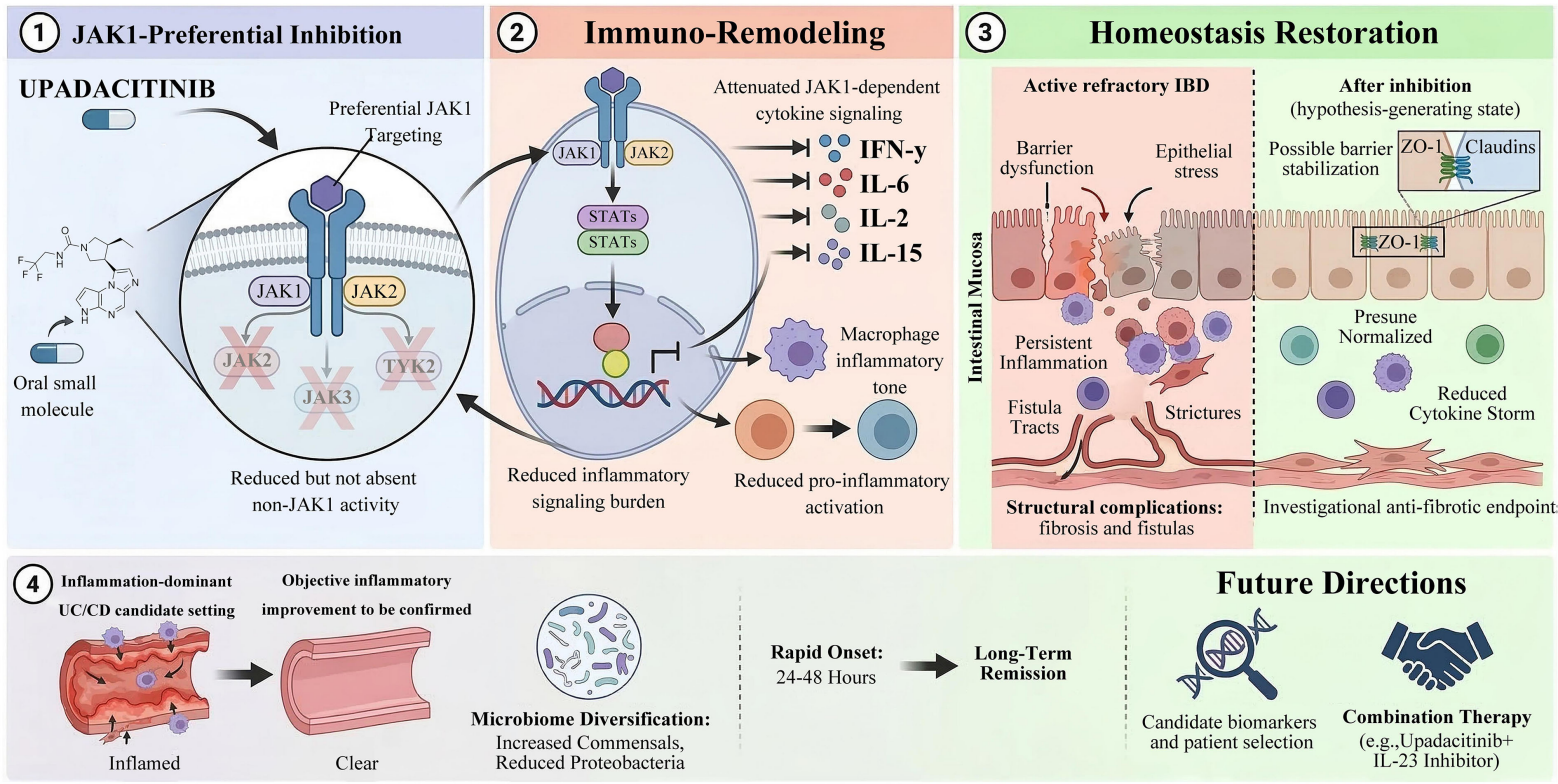
Conceptual model of rapid immune recalibration following JAK1-preferential inhibition in refractory inflammatory bowel disease. This conceptual figure illustrates upadacitinib as a JAK1-preferential inhibitor that may rapidly attenuate convergent inflammatory cytokine signaling in selected inflammation-dominant refractory IBD. Barrier stabilization, microbiome changes, anti-fibrotic endpoints, biomarker-guided selection, and combination therapy are presented as hypothesis-generating or investigational directions rather than established therapeutic consequences. Rapid symptom improvement should not be interpreted as proof of mucosal healing, transmural repair, fibrosis reversal, or disease modification without objective validation.

Thus, rapid immune recalibration should be interpreted as a hypothesis-generating construct rather than a validated predictive model (**Figure 1**). It is most relevant to selected inflammatory refractory IBD settings and should be tested prospectively against objective inflammatory and structural endpoints (4).

To avoid overstating the current evidence, the framework should be interpreted across three evidentiary levels. First, the established clinical evidence supports upadacitinib as an effective therapy for moderate-to-severe UC and CD, including clinical remission, endoscopic response, and early symptom improvement in trial and *post hoc* settings (5). Second, the mechanistic rationale is based on known JAK-STAT biology, including modulation of JAK1-dependent cytokine pathways and inflammatory amplification loops, but this does not by itself prove a validated recalibration state. Third, barrier stabilization, microbiome remodeling, anti-fibrotic potential, biomarker-guided selection, and combination strategies should be regarded as biologically plausible or hypothesis-generating directions rather than established therapeutic consequences of upadacitinib.

## 3 JAK1-preferential inhibition as rapid immune recalibration

JAK-signal transducer and activator of transcription (STAT) signaling functions as a central intracellular relay for many cytokines implicated in IBD. JAK1 participates in pathways downstream of interleukin (IL)-6, interferon-gamma, IL-2 family cytokines, IL-15, and other mediators that shape epithelial stress, innate immune activation, T-cell differentiation, and mucosal inflammatory persistence (6–8).

The pharmacological description of upadacitinib should be precise. Upadacitinib is more accurately described as JAK1-preferential rather than fully JAK1-selective, because JAK1 preference does not mean exclusive JAK1 activity in every tissue or at every exposure and does not eliminate class-related risk. Its relevance is that JAK1-preferential inhibition may prioritize inflammatory cytokine pathways while reducing, but not abolishing, interference with pathways more dependent on JAK2, JAK3, or TYK2. This distinction avoids two overstatements: that preferential activity guarantees safety, or that JAK inhibition is simply broad immunosuppression without mechanistic nuance.

This convergence is the biological basis for the proposed recalibration concept. In active refractory IBD, mucosal inflammation may be sustained by overlapping cytokine signals rather than by a single dominant mediator. Preferential inhibition of JAK1 can attenuate several inflammatory circuits simultaneously, including interferon responses, IL-6-driven STAT3 activation, lymphocyte activation, and macrophage inflammatory tone. The clinical implication is not simply broader suppression. It is the possibility of rapidly lowering inflammatory flux in patients whose disease is maintained by cytokine redundancy, a putative state that still requires prospective validation.

This framework is not intended to imply that rapid immune recalibration is unique to upadacitinib or exclusive to JAK1-preferential inhibition. Rather, upadacitinib provides a clinically relevant model because it has demonstrated efficacy in both UC and CD, including biologic-experienced populations, whereas other JAK inhibitors, including tofacitinib and filgotinib, differ in kinase selectivity, approved indications, availability, evidence base, and safety context. Rapid symptom improvement alone is therefore insufficient to establish a distinct disease-modifying paradigm; it should be treated as an early signal that requires objective confirmation.

## 4 Clinical signals supporting a recalibration model

The pivotal UC program provides strong clinical support for upadacitinib as a rapid and effective advanced therapy. In the U-ACHIEVE and U-ACCOMPLISH induction trials, and the U-ACHIEVE maintenance trial, upadacitinib improved clinical remission and endoscopic outcomes compared with placebo in patients with moderately to severely active UC (9). *Post hoc* analyses further suggested that UC symptoms may improve very early after treatment initiation, including diary-based symptom improvement during the first days of induction therapy (10). These observations are compatible with rapid suppression of inflammatory signaling, although symptom improvement alone cannot prove mucosal healing or disease modification.

In CD, the U-EXCEL and U-EXCEED induction trials and the U-ENDURE maintenance trial established efficacy of upadacitinib in patients with moderate-to-severe disease, including populations with previous biologic failure (11). Early symptom reduction during induction has also been reported in CD, supporting the view that JAK1-preferential inhibition may act quickly in selected inflammatory states (12). These data are particularly relevant because first-generation JAK inhibition with tofacitinib did not establish a comparable therapeutic role in CD, whereas JAK1-preferential inhibition has now shown efficacy across both major forms of IBD.

Comparative evidence and network meta-analyses suggest that upadacitinib is among the more potent currently available advanced therapies for induction in moderate-to-severe UC and has a clinically meaningful role in luminal CD (13, 14). However, a Perspective should resist translating comparative efficacy into a simplistic hierarchy. The more useful question is which inflammatory phenotype is most likely to benefit from rapid intracellular pathway modulation. Candidate features include high inflammatory burden, active cytokine signaling, rapid symptom turnover, biologic-experienced disease, and insufficient response to single-target biologic mechanisms. These features require prospective validation rather than retrospective inference.

## 5 Therapeutic positioning: from drug sequencing to phenotype-informed choice

The availability of multiple biologics and small molecules has improved IBD care, but it has also created a practical problem: treatment sequencing often remains empirical. Clinicians commonly choose the next agent according to prior drug exposure, safety profile, route of administration, comorbidities, and patient preference. These considerations are essential, but they do not fully capture the biological reason why a patient failed a previous therapy or why a rapidly acting intracellular inhibitor may be appropriate.

A recalibration framework suggests a more explicit positioning logic, but it should be linked to measurable criteria. Upadacitinib may be most relevant when the therapeutic goal is to quickly reduce active inflammatory signaling in refractory but still inflammation-dominant disease. Practical features include objective endoscopic inflammation, elevated C-reactive protein, elevated fecal calprotectin, imaging evidence of active inflammation in CD, high symptom burden, primary non-response or secondary loss of response to biologics, and a need for rapid steroid-sparing control.

Several groups require a more cautious threshold. Older patients, patients with cardiovascular or thrombotic risk factors, patients with previous malignancy, patients at high risk of serious infection or herpes zoster, and patients requiring combination or closely sequential advanced therapies need individualized benefit-risk assessment before JAK inhibitor use. In these settings, the attractiveness of rapid symptom control should not obscure baseline safety risk.

The framework should also specify where benefit should not be over expected. Fixed fibrostenotic disease, established strictures, chronic complex fistula anatomy, longstanding tissue damage, and disease states in which inflammation is no longer the dominant driver may require endpoints and interventions beyond cytokine suppression. For these patients, symptom improvement would not be sufficient evidence that structural disease has been modified (15).

The oral route also changes how positioning should be discussed. Oral delivery is not merely a convenience feature; it affects patient preference, treatment logistics, immunogenicity, and the feasibility of rapid initiation or adjustment. These practical advantages are especially relevant in patients who have already experienced infusion burden, injection fatigue, or immunogenicity to biologic therapy. Nevertheless, convenience should not drive use in isolation. In a mechanism-oriented framework, route of administration supports patient-centered implementation only after the inflammatory phenotype and safety profile have made JAK1-preferential inhibition a reasonable therapeutic option.

This positioning logic should remain evidence-linked. If JAK1-preferential inhibition is treated as a generic late-line option, response heterogeneity will remain difficult to interpret. If it is tested in selected inflammation-dominant settings, trials can evaluate whether early symptoms, biochemical markers, endoscopic activity, imaging findings, and exploratory molecular endpoints move together. Such designs would help separate rapid anti-inflammatory benefit from durable mucosal repair and structural disease modification.

## 6 Safety boundaries of JAK1-preferential inhibition

The same mechanism that makes upadacitinib attractive in refractory IBD also defines its safety boundary. Multi-pathway immunomodulation can reduce inflammatory signaling quickly, but it can also impair host defense and alter immune surveillance. Herpes zoster reactivation, acne, laboratory abnormalities, lipid changes, serious infection, and rare thrombotic or cardiovascular events must therefore be considered when selecting patients and monitoring treatment (16, 17).

Safety interpretation should be precise. Class warnings for JAK inhibitors were strongly influenced by the ORAL Surveillance trial of tofacitinib in rheumatoid arthritis, which enrolled older patients with cardiovascular risk factors and found higher incidences of major adverse cardiovascular events and malignancy compared with tumor necrosis factor inhibitors (18). These findings should not be ignored, but neither should they be applied mechanically to every IBD population or to every JAK inhibitor without attention to baseline risk, indication, age, dose, treatment duration, smoking status, prior malignancy, cardiovascular disease, and concomitant immunosuppression (19).

The proposed recalibration framework should not lower the threshold for JAK inhibitor use in patients with substantial baseline safety risks. Instead, rapid benefit must be paired with disciplined risk stratification. This includes vaccination assessment, especially for herpes zoster where appropriate; screening for infection risk; review of thrombotic and cardiovascular risk factors; lipid monitoring; and avoidance of unnecessary combination immunosuppression. In patients with high inflammatory burden and limited alternatives, the benefit-risk balance may favor a rapidly acting oral agent. In patients with substantial cardiovascular, thrombotic, malignancy, or infection risk, the threshold for use should be higher and the monitoring plan more explicit.

Dose and duration are also part of the safety boundary. The clinical meaning of a high-intensity induction phase differs from that of long-term maintenance exposure, and future analyses should avoid collapsing these periods into a single safety estimate. A practical clinical framework would distinguish short-term induction benefit, objective evidence of response, transition to the lowest effective maintenance strategy, and periodic reassessment of ongoing need. This is particularly important if upadacitinib is considered after multiple prior therapies or in patients who may also require corticosteroids, immunomodulators, or biologic agents.

## 7 Future directions: biomarkers, structural endpoints, and prospective testing

The most important research priority is to identify who is experiencing proposed immune recalibration rather than nonspecific symptomatic improvement. For practical clarity, prospective testing should distinguish clinical variables, routine inflammatory biomarkers, objective disease-assessment endpoints, and exploratory translational endpoints.

Clinical variables may include prior biologic failure pattern, primary non-response or secondary loss of response, corticosteroid requirement, stool frequency, rectal bleeding, abdominal pain, high symptom burden, and early symptom trajectory during induction. Routine inflammatory biomarkers include C-reactive protein and fecal calprotectin, which can provide feasible biochemical evidence of inflammatory change in clinical practice. Objective disease-assessment endpoints should include endoscopic activity, histologic activity in UC, and MRI or intestinal ultrasound evidence of inflammation and transmural response in CD. These measures are essential for distinguishing rapid symptomatic improvement from objective disease control (20).

Exploratory translational endpoints should be separated from markers that are already clinically feasible. Mucosal transcriptomics, phospho-STAT activity, cytokine panels, single-cell profiling, microbiome and metabolome profiling, and epithelial barrier assays may help test the biological basis of the recalibration model, but they should be presented as research tools rather than routine clinical selection criteria.

Prospective studies could test the framework by stratifying patients at baseline according to inflammatory burden and prior treatment-failure pattern, then measuring early symptom and biomarker changes at weeks 2, 4, and 8. These early trajectories should be linked to week 8 or week 12 endoscopic response, histologic improvement in UC, imaging or transmural outcomes in CD, and durable remission during maintenance (21). Such designs would help separate rapid anti-inflammatory benefit from mucosal healing, transmural repair, and structural disease modification.

Barrier and microbiome endpoints should be treated as mechanistically plausible but not yet definitive. Active inflammation disrupts epithelial barrier function and is associated with microbial dysbiosis, and reducing inflammatory stress may secondarily influence both processes (22, 23). However, whether upadacitinib directly restores barrier integrity or produces clinically meaningful microbiome remodeling remains uncertain. Future studies should distinguish direct drug effects from secondary changes caused by reduced inflammation.

Structural complications require particular caution. Fibrosis and fistulas remain major unmet needs in CD, and inflammatory pathways may interact with fibroblast activation, extracellular matrix deposition, and tissue remodeling (24). Nevertheless, established strictures and complex fistula tracts are not simply cytokine-driven inflammatory lesions. Claims about anti-fibrotic or fistula-healing effects of JAK1 inhibition should therefore be framed as hypotheses requiring dedicated endpoints, imaging, surgical correlation, and long-term follow-up.

Combination strategies, including dual targeted therapy, are another frontier for highly refractory IBD. Combining a rapidly acting small molecule with a biologic that targets a complementary pathway may be attractive in selected patients, but the evidence base remains limited and safety must be central (25). A rational approach would reserve combination therapy for carefully selected refractory cases, define stopping or de-escalation rules, and collect prospective safety and effectiveness data. In this context, upadacitinib should not be presented as an unrestricted partner for combination therapy, but as a candidate component of mechanism-guided regimens whose risks remain to be defined.

## 8 Discussion

Upadacitinib has expanded the therapeutic landscape of IBD, but its conceptual value should be interpreted with appropriate evidentiary boundaries. By inhibiting JAK1-dependent signaling downstream of multiple cytokines, it highlights a treatment logic that differs from sequential single-target biologic switching. The recalibration framework proposed here interprets rapid clinical benefit as a signal that some refractory inflammatory states may be maintained by convergent intracellular cytokine signaling that can be pharmacologically modulated, at least transiently.

This interpretation has several practical implications. First, upadacitinib should be positioned through both clinical and biological criteria, not only through prior treatment sequence. Second, early symptom improvement should be connected to objective inflammatory endpoints before being interpreted as meaningful disease control. Third, safety should be integrated into the positioning logic rather than treated as a separate checklist, because rapid multi-pathway immunomodulation creates both opportunity and risk. Finally, future studies should test whether biomarkers can prospectively identify patients with putative cytokine-driven inflammatory states who are most likely to benefit.

The main limitation of this Perspective is that the recalibration model is inferential. Therefore, it should be used to organize clinical reasoning and prospective testing rather than to claim an established biological mechanism or durable immune reset. It is supported by known JAK-STAT biology and by clinical observations of rapid efficacy, but it has not yet been validated as a predictive framework. Cytokine-dominant disease is not an established clinical phenotype, and early symptom improvement cannot prove mucosal healing, transmural repair, fibrosis reversal, or durable disease modification. Barrier stabilization, microbiome remodeling, anti-fibrotic potential, biomarker-guided selection, and combination therapy should therefore remain hypothesis-generating directions until tested prospectively.

A second limitation is that refractory IBD is clinically heterogeneous. The framework is most applicable to selected inflammatory settings, especially when objective biomarkers, endoscopy, histology, or imaging show active inflammation. It should not be generalized to fixed structural complications or to patients whose dominant problem is safety risk rather than uncontrolled inflammatory burden. If prospectively validated, a recalibration framework could help move refractory IBD management from empirical drug switching toward mechanism-informed therapeutic choice while preserving a cautious distinction between rapid response and disease modification.
